# Prevalence of dry eye disease among Chinese high school students during the COVID-19 outbreak

**DOI:** 10.1186/s12886-022-02408-9

**Published:** 2022-04-26

**Authors:** Feng Lin, Yuying Cai, Xinfeng Fei, Yimin Wang, Minwen Zhou, Yan Liu

**Affiliations:** 1 Department of Ophthalmology, Shanghai General Hospital, Shanghai Jiaotong University School of Medicine, Shanghai, China; 2 Eye Institute and Department of Ophthalmology, Eye & ENT Hospital, Fudan University, Shanghai, China; 3 National Clinical Research Center for Eye Diseases, Shanghai, China; 4 Shanghai Key Laboratory of Ocular Fundus Diseases, Shanghai, China; 5 Department of Ophthalmology, Yangpu Hospital, School of Medicine, Tongji University, Shanghai, China; 6 Department of Ophthalmology, Shanghai Fourth People’s Hospital Affiliated to Tongji University School of Medicine, Shanghai, China

**Keywords:** Dry eye disease, Prevalence, High school, Risk factors, COVID-19

## Abstract

**Purpose:**

The study aimed to investigate the prevalence of dry eye disease (DED) and relevant risk factors among Chinese high school students during the COVID-19 outbreak.

**Methods:**

A cross-sectional study was conducted from November to December 2020, and 4825 high school students from nine high schools in Shanghai were recruited. All students completed ocular surface disease index (OSDI) and perceived stress scale (PSS) questionnaires and answered other questions designed to ascertain information on the risk factors related to DED. DED was diagnosed when OSDI scores were greater than or equal to 13. The prevalence of symptomatic DED was determined. A T-test, Kruskal-Wallis test, Chi-square test, and logistic regression analysis were used to examine the possible risk factors.

**Results:**

The prevalence of symptomatic DED among Chinese high school students was 70.5%. In univariate analysis, higher PSS scores (*P*<0.001), prolonged video display terminal (VDT) use (*P*<0.001), wearing contact lenses (*P*=0.001), poor sleep quality (*P*<0.001), and being female (*P*<0.001) were significantly correlated with dry eyes. In multivariate logistic regression analysis, higher PSS scores (*P*<0.001, OR=1.20), prolonged VDT use (*P*<0.001, OR=1.07), poor sleep quality (*P*<0.001, OR=1.84), and being female (*P*=0.001, OR=1.25) were significant risk factors associated with DED.

**Conclusions:**

Due to the epidemic, most Chinese high school students are in a high-risk environment in which they are more likely to suffer from DED, such as long online courses and heavy stress from school. Relevant preventive measures that may have a positive impact on public health and quality of life for high school students should be brought to the forefront.

**Supplementary Information:**

The online version contains supplementary material available at 10.1186/s12886-022-02408-9.

## Introduction

Dry eye disease (DED) is a condition occurring worldwide that is defined as a disorder of the tear film due to tear deficiency or excessive tear evaporation [1]. It could cause damage to the interpalpebral ocular surface and is correlated with symptoms of ocular discomfort [1]. Many epidemiological surveys regarding DED have been conducted around the world [2]. However, most of these surveys involved relatively older populations, over 40 years of age, and few population-based DED studies focused on high school students [2]. A population-based study investigating the prevalence of DED among Chinese college students revealed that the prevalence rate of DED reached 66.9 % [3]. DED is particularly common in the young population in China [3, 4]. In addition, due to the coronavirus disease outbreak in 2019 (COVID-19), online courses have become much more prevalent among high school students [5]. As excessive use of screens is one of the major causes of DED, students having long online courses may suffer from DED [6, 7]. Therefore, the prevalence of DED among high school students should not be neglected. It is necessary to conduct a preliminary survey on DED prevalence among Chinese high school students during the COVID-19 outbreak. In addition, considering the severe consequences of DED, such as keratitis, corneal ulcers, or even corneal perforation, early prevention of DED is important. By identifying significant risk factors, relevant preventive measures against DED could be implemented among high school students.

In this study, the prevalence and risk factors related to DED were analyzed among Chinese high school students who had taken online courses for at least half year at home and just returned to school.

## Methods

### Sample Size Calculation

A pre-survey was conducted in a randomly selected high school (323 subjects were involved). The prevalence rate of DED was 73.07%. The formula n = Z^2^p(1-p)/δ^2^ was used in the study (here, α=0.05, Z=1.96, *p=*73.07%, δ=0.02). Additionally, a nonresponse rate of 5% was considered. Therefore, a minimum of 1990 subjects was required in this study.

### Sampling and Survey

A cross-sectional survey was conducted among public high schools in Hongkou District in Shanghai from November to December 2020. Letters were sent to the principals of all 11 public high schools (according to the public high school list published by Hongkou District Education Bureau; http://www.shhk.gov.cn/hkjy/) located in Hongkou district in Shanghai. The letter explained the purpose of the study. Finally, nine public high schools consented to the study. All students in the nine schools were recruited. A total of 4825 subjects were included in our survey. Written informed consent was obtained from all subjects and parents of subjects of age group less than 16. The study was approved by the Ethics Committee of Shanghai General Hospital and adhered to the tenets of the Declaration of Helsinki (NO. 2020KY026).

### OSDI Questionnaire

The ocular surface disease index questionnaire (OSDI questionnaire) is one of the most widely used questionnaires to evaluate DED [8–11]. It is viewed as one of the gold standards regarding DED according to the Dry Eye Work Shop (DEWS) consensus [12]. In this study, a simplified Chinese version of the OSDI questionnaire, whose reliability and validity was previously verified, was utilized [13]. It was divided into three dimensions: ocular symptoms, vision-related function, and environmental trigger scores. The ocular symptoms score consisted of three items, namely “eyes that are sensitive to light,” “eyes that feel gritty,” and “painful or sore eyes.” The vision-related function score consisted of five items: “blurred vision,” “poor vision,” “reading,” “driving at night,” “working with a computer or bank machine,” and “watching TV.” The environmental triggers score consisted of three items: “windy conditions,” “places or areas with low humidity,” and “areas that are air conditioned.” The severity was graded on a scale of 0 to 5 according to the frequency regarding each item, which represented “none of the time,” “some of the time,” “half of the time,” “most of the time,” and “all the time.” The OSDI score was based on the formula: [(sum of scores for all questions answered) *100] / [(total number of answered questions) *4]. Using the formula, the total OSDI score could be calculated.

### Definition of DED

According to the total OSDI score, DED was considered as “symptomatic DED” and was defined as a subject whose OSDI score was greater than or equal to 13 [14, 15]. Additionally, subjects were classified into 4 categories by OSDI score: normal (scores 0–12), mild DED (13–22), moderate DED (23–32), and severe DED (33–100 ) [14].

### Definition of risk factors

In total, five questions were related to potential risk factors, namely “psychological stress,” “VDT use,” “wearing contact lenses,” “sleep quality,” and “gender.” To measure and evaluate psychological stress, the Chinese version of the perceived stress scale (PSS) was utilized. The PSS questionnaire is one of the most widely used psychological instruments for measuring the perception of stress. Developed by Cohen in 1983, it has shown sufficient reliability and validity [16]. The Chinese version of the PSS questionnaire was also found to be reliable [17, 18]. Originally, it consisted of 14 items that were designed to determine how unpredictable, uncontrollable, and overloaded respondents find their lives. To facilitate the implementation, the four items version of PSS was adapted for this study. This version consisted of four PSS items, two of which were negative (“how often have you felt that you were unable to control the important things in your life” and “how often have you felt difficulties were piling up so high that you could not overcome them”), while the other two items were positive (“how often have you felt confident about your ability to handle your personal problems” and “how often have you felt that things were going your way”). The subjects were required to answer each question using a five-point Likert scale score ranging from 0 (never) to 4 (very often) and report the event frequency correlated with the PSS items in the last month. PSS scores were obtained by reversing responses (0 = 4, 1 = 3, 2 = 2, 3 = 1, 4 = 0) to the two positively stated items and summing all scale items. The total scores ranged from 0 to 16, and subjects with higher scores had higher psychological stress levels. The video display terminal (VDT) use question was defined as “the average hours spent on VDT devices, such as iPad, iPhone, or computers, per day for the last week.” The wearing contact lenses question was defined as wearing a contact lens at least once a week for the last three months. Poor sleep quality was defined as difficulty in falling asleep.

### Research process

Before the formal survey began, the project leader trained project members and standardized the survey process. On the day of the survey, the subject had to fill out a questionnaire that consisted of four parts (Supplemental Figure 1):

(1) General information: name, gender, age, school, class, and student identification number

(2) OSDI questionnaire

(3) DED risk factors (contact lens wear, sleep quality and VDT use) and medical history regarding eye diseases (self-reported ocular inflammation, such as conjunctivitis and keratitis, or previous ocular surgery history within the last 6 months)

(4) PSS questionnaire

Questionnaires with omissions were considered invalid. Two project members transferred the data to a database independently. Subjects who had a history of any ocular inflammation or ocular surgery within the last six months were excluded from the survey.

### Statistical Analyses

The normality of the data distribution was tested using the Kolmogorov–Smirnov test. Normal distribution parameters were expressed as the mean (standard deviation, SD). Non-normal distribution parameters were expressed as the median (interquartile range, IQR). The prevalence of DED was calculated as a ratio. In univariate analysis, normally distributed continuous variables were compared by a T-test, while non-normally distributed continuous variables were compared by the Mann-Whitney U test. Chi-square tests were used for comparisons between categorical variables. All risk factors were then introduced into a binary logistic regression model. Relative risks were estimated as odds ratios (OR) with a 95% confidence interval (CI). *P*<0.05 was regarded as a significant difference. Data analyses were carried out using SPSS version 21.0 (SPSS, Chicago, IL, USA).

## Results

In this study, a total of 4722 valid questionnaires were included. The questionnaire reclaiming efficiency was 97.9%. Among the 4722 subjects in our selected schools, 28 subjects were excluded because of ocular inflammation or ocular surgery. A flowchart pertaining to the study is shown in Supplemental Figure 2.

In total, 4694 subjects were included in our survey, including 2248 males and 2446 females. The mean age was 16.39 (0.95), which ranged from 14 to 19. Detailed characteristics regarding the subjects are summarized in Table 1.

**Table 1:** Subjects' characteristics

High school students	n (%)
Gender	
Male	2248 (47.9)
Female	2446 (52.1)
Age	
14	12 (0.3)
15	870 (18.5)
16	1720 (36.6)
17	1482 (31.6)
18	585 (12.5)
19	25 (0.5)
Grade	
Grade10	1662 (35.4)
Grade11	1568 (33.4)
Grade 12	1464 (31.2)

**Fig. 1:**
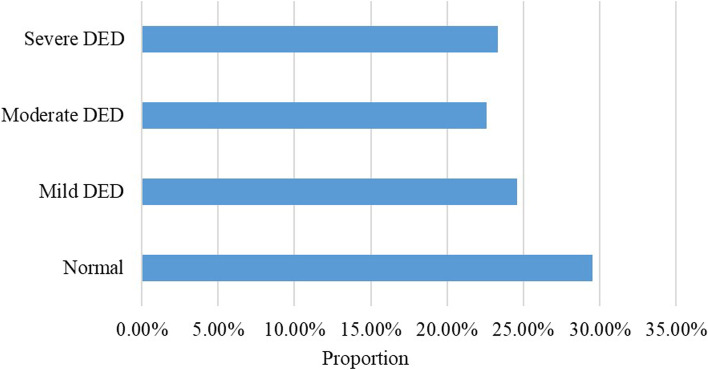
The distribution of OSDI scores

Symptomatic DED was present in 3311 subjects. The prevalence of symptomatic DED was 70.5%. According to the OSDI scores, all DED subjects were then divided into three groups, in which 1154 subjects reported mild ocular surface symptoms (13–22), 1063 subjects reported moderate ocular surface symptoms (23–32), and 1094 subjects reported severe ocular surface symptoms (33–100). The distribution of OSDI scores is presented in Figure 1.

**Fig. 2:**
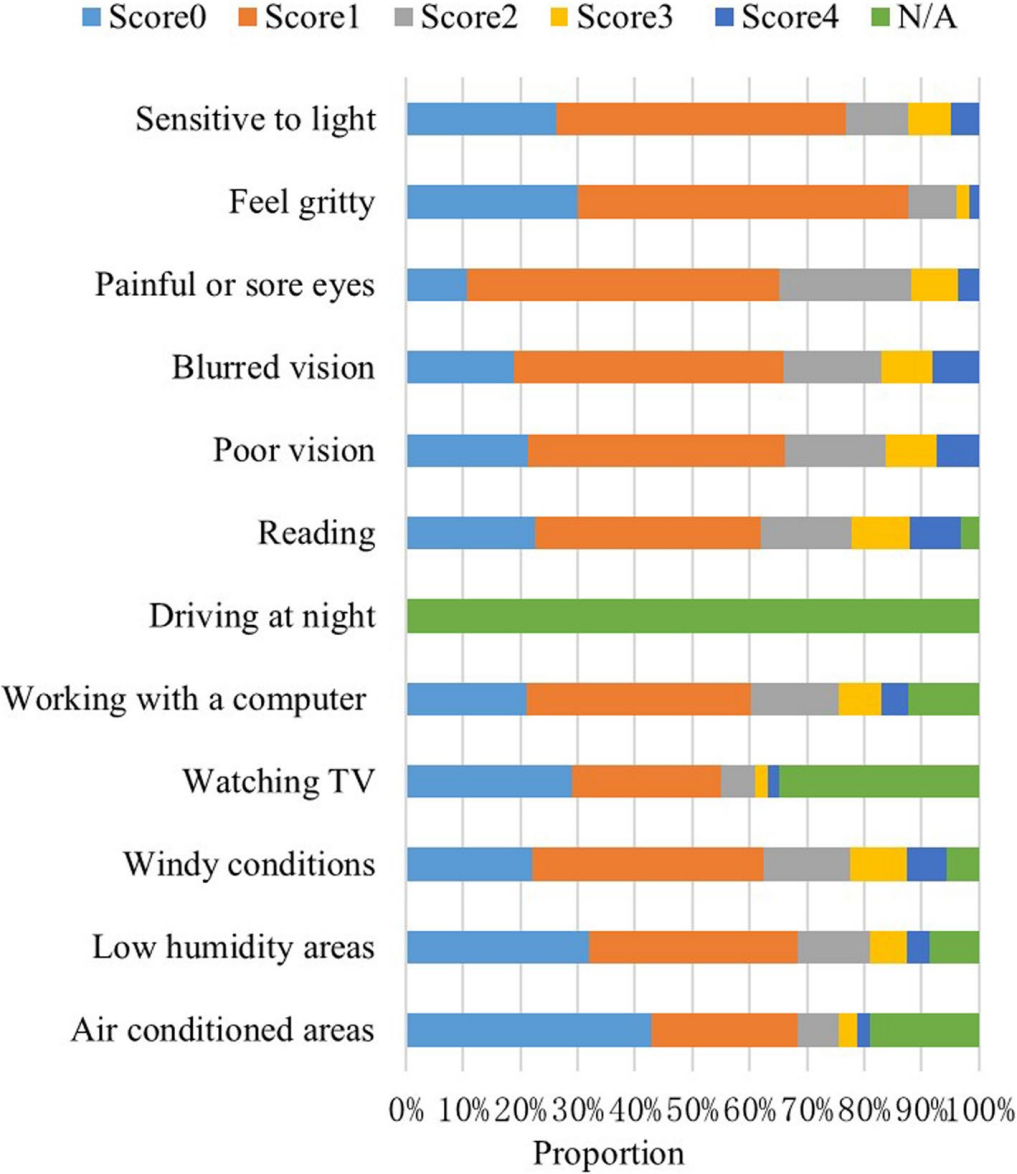
OSDI score by item in DED subjects Each question ranged from 0 to 4 according to the frequency (0=none of the time, 1=some of the time, 2=half of the time, 3=most of the time, 4=all of the time for the last week). N/A meant the individual did not perform the task or stay in the situation for the last week

Among the 12 items from the OSDI questionnaire in 3311 DED subjects, the most frequently (item score ≥ 1) reported ocular symptom was painful or sore eyes (89.3%). The most frequently (item score ≥ 1) reported vision-related function problem was blurred vision (81.1%). The most frequently (item score ≥ 1) reported environmental trigger was windy conditions (72.3%). The distribution of the scores in the 12 items from the OSDI questionnaire is presented in Figure 2.

In the univariate analysis, PSS scores were higher in DED subjects than in normal subjects (*P*<0.001). Additionally, the DED group had a longer VDT use time (*P*<0.001) (Supplemental Table 1). Wearing contact lenses (*P*=0.012), poor sleep quality (*P*<0.001), and being female (*P*<0.001) were significantly correlated with DED (Supplemental Table 2).

Table 2 shows the binary logistic regression model. DED subjects had higher PSS scores (OR=1.20, *P*<0.001) and spent more time on VDT devices (OR=1.07, *P*<0.001). Poor sleep quality (OR=1.84, *P*<0.001) and being female (OR=1.25, *P*=0.001) were still risk factors related to DED. Although wearing contact lenses was related to DED in univariate analysis, it was not a risk factor significantly related to DED in the binary logistic regression analysis (*P*=0.152).

**Table 2:** Binary logistic regression analysis of risk factors for DED

Risk Factors	OR (95%CI)	*P*
PSS score	1.20 (1.17-1.24)	*P*<0.001
VDT use time	1.07 (1.03-1.10)	*P*<0.001
Contact lens wear		
Yes	1.19 (0.94-1.50)	*P*=0.152
No	1.00 (Ref)	
Poor sleep quality		
Yes	1.84 (1.57-2.16)	*P*<0.001
No	1.00 (Ref)	
Gender		
Female	1.25 (1.09-1.43)	*P*=0.001
Male	1.00 (Ref)	

## Discussion

The current study found that the prevalence of symptomatic DED among Chinese high school students was 70.5%. In comparison with other DED epidemiological surveys that also adopted the same DED diagnostic criteria based on OSDI questionnaires, a study focusing on the prevalence of DED among Mexican high school students revealed that 65.3% of the students suffered from symptomatic DED [10]. Another cross-sectional survey among Brazilian undergraduate students revealed that 59.6% of totally 2140 subjects scored greater than 12 based on OSDI questionnaires [11]. Still another cross-sectional study among 650 undergraduate students in Ghana revealed that the prevalence of DED based on OSDI questionnaires was 48.1 % [9]. The prevalence reported in our study was higher than that in the above-mentioned three studies. Considering the high prevalence of DED among Chinese high school students in the current study, we speculated that it may attribute to long online courses and heavy stress. For one thing, high school students may have longer and more frequent online courses affected by the pandemic than before. For another thing, high school students may have heavy psychological stress especially during the pandemic. Due to the emphasis on academic performance, the positive rate of mental health problems among Chinese high school students was quite high [19]. In addition, long online courses during the COVID-19 lockdown may increase stress for students [20].

As for other epidemiological surveys on DED among high school students, Zhang et al. [4] conducted an epidemiological survey amongst Chinese high school students in 2010 that revealed that the prevalence of symptomatic DED was 23.7%. In contrast, Uchino et al. [21] reported that the prevalence of symptomatic DED among Japanese high school students was 21.6% in 2008. Both studies adopted the Schaumberg questionnaire [22] as the DED diagnostic tool and showed similar prevalence rates of DED among high school students. As the prevalence rate they reported was far lower than that in this study, it may be attributed to different DED diagnostic tools.

Among all OSDI questionnaire items, blurred vision was the most frequently reported vision-related function problem by DED subjects. This could be due to ocular surface damage in the overlying optical zone, which could cause higher-order aberrations and increased corneal backward light scattering [23]. Additionally, it was difficult to detect visual or optical changes using standard visual acuity testing. As student vision screening *is conducted annually in high schools, some students may complain about blurred vision though their vision acuity is good.* The PSS questionnaire is one of the most widely used psychological instruments for measuring the perception of stress [18]. The interactive relationship between psychological stress and DED has been reported in many studies. [24–26] High psychological stress could lead to DED [24, 26]. Inversely, with the pain and discomfort caused by tear film instability, it could contribute to psychosomatic symptoms [25, 27].

In the current study, we also identified that high psychological stress may induce DED. However, the pathophysiology of how psychological stress induced DED has not been fully elucidated. It was reported that stress may induce depression [28]. In depressed subjects, disturbances of serotonin receptors, located around the conjunctival epithelium, could influence meibomian glands, which can lead to tear film deficiency [29, 30]. In addition, heavy psychosocial stress may reduce pain modulation capabilities [31]. Subjects with heavy stress may show significant DED symptoms even with only minor changes in the tear film stability. It was worth noting that the high PSS scores among Chinese high school students (median score in DED subjects: 8.00, in non-DED subjects: 6.00) were comparable to those of medical students [26]. This may be attributed to the fact that students under East Asian culture are more likely to show higher depression and lower life satisfaction than their European counterparts due to the emphasis on academic performance [32].

Prolonged VDT use has been viewed as a risk factor related to DED in many studies [33, 34], which was corroborated in this study. Cumulative years of VDT use may change lacrimal gland function and ultimately result in decreased tear secretion [35]. Furthermore, prolonged VDT users have shorter tear film break-up time [33], which may contribute to prolonged blinking intervals while gazing at VDT devices [36]. A diminished blink frequency rate and incomplete blinking could also contribute to DED [36]. As the use of iPads, iPhones, and laptops is much more common than before, especially in young teenagers [37], the high risk of prolonged VDT use causing DED should be highlighted.

As a risk factor related to DED in this study, several studies have demonstrated a correlation between sleep quality and DED [38–40]. Sleep deprivation could disrupt the lacrimal system and induce DED [39]. With poor sleep quality, parasympathetic tone can be reduced, as removal of parasympathetic innervation to the lacrimal gland can lead to a rapid reduction in tear flow and induced DED [40]. Additionally, a high proportion of poor sleep quality (32.53%) among all high school students was noted in our study. This could be due to the fact that Chinese high school students often have a heavy workload and fierce competition for university entrance and parents’ high expectations of their academic achievement [41].

Females showed a 1.25-fold higher risk of having DED than males. Being female has been identified as a significant risk factor for the development of DED in many studies [10, 42, 43], which could largely be attributed to the effects of sex steroids, such as androgens and estrogens. Females have unique hormonal cycles (menstruation, pregnancy, and menopause) that can affect tear film stability. For example, a study showed that a low androgen level was a consistent risk factor for DED [43]. Although the association of sex steroids with DED has not been fully determined, their influence should be considered.

Concerning the significantly correlated risk factors found in this study, taking precautions is necessary to reduce the prevalence of DED. For example, students’ mental status should be taken seriously, and school staff should appropriately alleviate students’ academic pressure. Consolation or even psychological counseling performed by school doctors for students who have poor sleep quality and heavy stress is recommended. In addition, students should be persuaded to reduce VDT use time. A one-minute break for every 30 minutes of VDT use or deliberately excessive blinking is highly recommended.

Wearing contact lenses has been viewed as a risk factor related to DED in many studies [21, 44]. When a contact lens is placed on the ocular surface, the tear film becomes separated into the pre- and post-lens tear film. Because the tear film becomes separated, the volume of the aqueous layer at the pre-lens tear film is decreased and becomes unstable and easily breakable within a short period after blinking [45]. However, it was not a significant risk factor related to DED in this study. This might be due to the different proportions of subjects wearing contact lenses. Compared with an epidemiological survey among Japanese high school students, 37.8% of students wore contact lenses, while only 9.71% of students used contact lenses in this study.

This study has some limitations and biases. First, only nine high schools in Shanghai were included in our survey. Schools in other areas in China were not covered in this study. Although this does not rule out selection bias, it is still the largest population-based epidemiological survey among Chinese high school students. This study had a large sample size as it was well over that recommended by statistical tests. Second, other risk factors related to DED, such as diabetes and smoking, were not analyzed in the study [46, 47]. However, the prevalence rate of diabetes among high school students is pretty low, and smoking is forbidden in Chinese high schools. Finally, our study lacked the parameters of dry eye signs, and the prevalence of DED could vary from different diagnostic criteria. Only the prevalence of symptomatic DED based on the OSDI questionnaire was reported in this study. Further study should incorporate objective dry eye examinations to further validate the results from the symptom questionnaires.

## Conclusion

In summary, this cross-sectional study demonstrated a fairly high prevalence rate of DED among Chinese high school students during the COVID-19 outbreak for the first time. In addition, there were some significant risk factors, such as increased psychological stress, prolonged VDT use, poor sleep quality, and being female. As students were affected by the pandemic, most Chinese high school students are in a high-risk environment in which they may suffer from DED, such as long online courses and heavy stress from school. Relevant preventive measures that may have a positive impact on public health and quality of life for high school students should be brought to the forefront.

## Supplementary Information

**Additional file 1.**

**Additional file 2.**

**Additional file 3.**

**Additional file 4.**

## Data Availability

Dataset analyzed in the current study are available in the supplemental materials which is a spreadsheet named “Data”.
