# Supplementary material for: Prevalence of dry eye disease among Chinese high school students during the COVID-19 outbreak
Source: BMC Ophthalmol. 2022 Apr 26;22:190. doi: 10.1186/s12886-022-02408-9 (PMC9038515; doi:10.1186/s12886-022-02408-9)
Supplement: Supplementary file 1 — Additional file 1. [file 12886_2022_2408_MOESM1_ESM.pdf]

Part I General information

School： ____________ Class： ____________ Student ID： ____________

Name： _____________ Gender： __________ Age： ___________

Part II Ocular surface disease index (OSDI)

1. **Have you experienced any of the following during the last week?**

	All the time	Most of the time	Half of the time	Some of the time	None of the time
Eyes that are sensitive to light	4	3	2	1	0
Eyes that feel gritty	4	3	2	1	0
Painful or sore eyes	4	3	2	1	0
Blurred vision	4	3	2	1	0
Poor vision	4	3	2	1	0

2. **Have problems with your eyes limited you in performing any of the following during the last week?**

	All the time	Most of the time	Half of the time	Some of the time	None of the time	
Reading	4	3	2	1	0	N/A
Driving at night	4	3	2	1	0	N/A
Working with a computer	4	3	2	1	0	N/A
Watching TV	4	3	2	1	0	N/A

3. **Have your eyes felt uncomfortable in any of the following situations during the last week?**

	All the time	Most of the time	Half of the time	Some of the time	None of the time	
Windy conditions	4	3	2	1	0	N/A
Places or areas with low humidity (very dry)	4	3	2	1	0	N/A
Areas that are air conditioned	4	3	2	1	0	N/A

Part III Risk factors and medical history

1.	Wearing a contact lens at least once a week for the last three months	Yes	No
2.	Difficulty in falling asleep	Yes	No
3.	Ocular surgery history within the last 6 months	Yes : __________	No
4.	Ocular inflammation	Yes : __________	No
5.	The average hours spent on VDT devices, such as iPad, iPhone, or computers, per day for the last week	__________hours	

Part IV Perceived stress scale (PSS)

1.	In the last month, how often have you felt that you were unable to control the important things in your life?<br>0 - never 1 - almost never 2 - sometimes 3 - fairly often 4 - very often
2.	In the last month, how often have you felt confident about your ability to handle your personal problems?<br>0 - never 1 - almost never 2 - sometimes 3 - fairly often 4 - very often
3.	In the last month, how often have you felt that things were going your way ?<br>0 - never 1 - almost never 2 - sometimes 3 - fairly often 4 - very often
4.	In the last month, how often have you felt difficulties were piling up so high that you could not overcome them ?<br>0 - never 1 - almost never 2 - sometimes 3 - fairly often 4 - very often

Supplemental Figure 1. Research questionnaire

The questionnaire consisted of four parts : (1) General information (2) Ocular surface disease index (3) Risk factors and medical history (4) Perceived stress scale
