# Supplementary figures and images for: Prevalence of dry eye disease among Chinese high school students during the COVID-19 outbreak
Source: BMC Ophthalmol. 2022 Apr 26;22:190. doi: 10.1186/s12886-022-02408-9 (PMC9038515; doi:10.1186/s12886-022-02408-9)

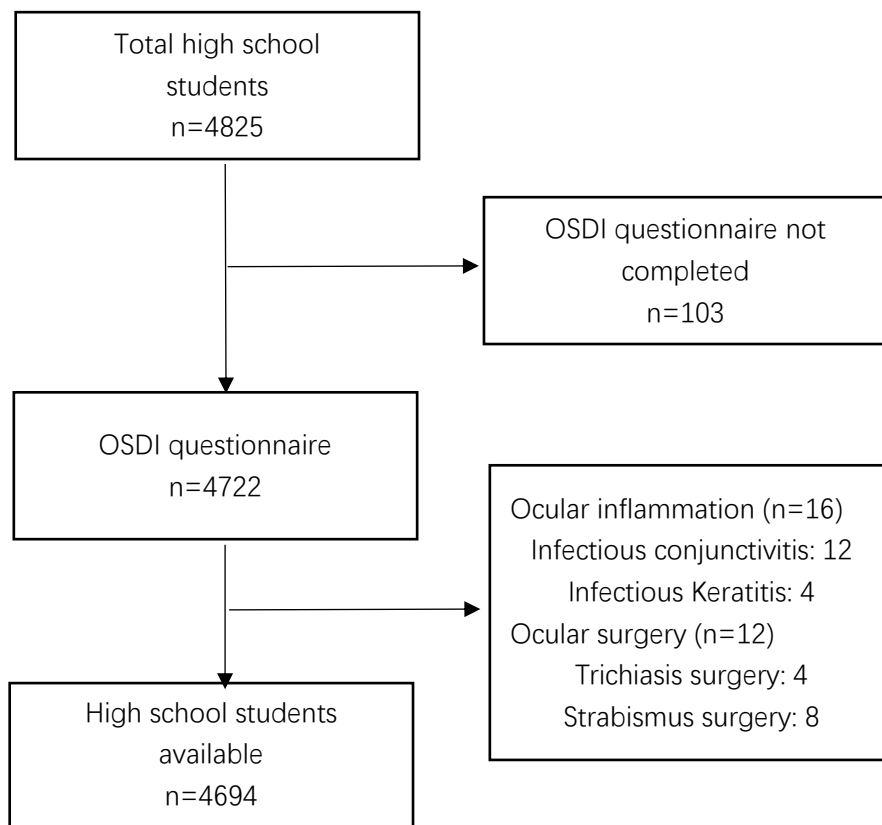

**Supplemental Figure 2. Workflow of the study**

Supplement: Supplementary file 2 — Additional file 2. [file 12886_2022_2408_MOESM2_ESM.pdf]
