# Supplementary material for: Prevalence of dry eye disease among Chinese high school students during the COVID-19 outbreak
Source: BMC Ophthalmol. 2022 Apr 26;22:190. doi: 10.1186/s12886-022-02408-9 (PMC9038515; doi:10.1186/s12886-022-02408-9)
Supplement: Supplementary file 3 — Additional file 3. [file 12886_2022_2408_MOESM3_ESM.docx]

| **Supplemental Table 1. Univariate analysis of risk factors for DED (continuous variables)** | | | | |
| --- | --- | --- | --- | --- |
| Risk Factors | Symptomatic DED  Yes (n=3311) | No (n=1383) | *Z* | *P* |
| PSS score | 8.00 (6.00-9.00) | 6.00 (4.00-8.00) | 18.41 | *P<*0.001 |
| VDT use time | 2.00 (1.00-3.00) | 2.00 (1.00-3.00) | 7.32 | *P*<0.001 |
