# Supplementary material for: Prevalence of dry eye disease among Chinese high school students during the COVID-19 outbreak
Source: BMC Ophthalmol. 2022 Apr 26;22:190. doi: 10.1186/s12886-022-02408-9 (PMC9038515; doi:10.1186/s12886-022-02408-9)
Supplement: Supplementary file 4 — Additional file 4. [file 12886_2022_2408_MOESM4_ESM.docx]

| **Supplemental Table 2. Univariate analysis of risk factors for DED (categorical variables)** | | | | |
| --- | --- | --- | --- | --- |
| Risk Factors | Symptomatic DED  Yes (N=3311), n | No (N=1383), n | χ2 | *P* |
| Contact lens wear |  |  |  |  |
| Yes | 345 | 111 | 6.37 | *P*=0.012 |
| No | 2966 | 1272 |  |  |
| Poor sleep quality |  |  |  |  |
| Yes | 1253 | 274 | 144.51 | *P*<0.001 |
| No | 2058 | 1109 |  |  |
| Gender |  |  |  |  |
| Male | 1508 | 740 | 24.78 | *P*<0.001 |
| Female | 1803 | 643 |  |  |
